# Characterization of ATPase Activity of P2RX2 Cation Channel

**DOI:** 10.3389/fphys.2016.00186

**Published:** 2016-05-24

**Authors:** Rahul Mittal, M'hamed Grati, Miloslav Sedlacek, Fenghua Yuan, Qing Chang, Denise Yan, Xi Lin, Bechara Kachar, Amjad Farooq, Prem Chapagain, Yanbin Zhang, Xue Z. Liu

**Affiliations:** ^1^Department of Otolaryngology, University of Miami Miller School of MedicineMiami, FL, USA; ^2^Laboratory of Cell Structure and Dynamics, Section on Structural Cell Biology, National Institute on Deafness and Other Communication Disorders, National Institutes of HealthBethesda, MD, USA; ^3^Department of Biochemistry, University of Miami Leonard M. Miller School of MedicineMiami, FL, USA; ^4^Department of Otolaryngology, Emory UniversityAtlanta, GA, USA; ^5^Department of Physics, Florida International UniversityMiami, FL, USA; ^6^Department of Otolaryngology, Central South University, Xiangya HospitalChangsha, China

**Keywords:** P2X2, ATPase activity, Ligand-gated ion channels, Electrophysiology, computer modeling

## Abstract

P2X purinergic receptors are plasma membrane ATP-dependent cation channels that are broadly distributed in the mammalian tissues. P2RX2 is a modulator of auditory sensory hair cell mechanotransduction and plays an important role in hair cell tolerance to noise. In this study, we demonstrate for the first time *in vitro* and in cochlear neuroepithelium, that P2RX2 possesses the ATPase activity. We observed that the P2RX2 V60L human deafness mutation alters its ability to bind ATP, while the G353R has no effect on ATP binding or hydrolysis. A non-hydrolysable ATP assay using HEK293 cells suggests that ATP hydrolysis plays a significant role in the opening and gating of the P2RX2 ion channel. Moreover, the results of structural modeling of the molecule was in agreement with our experimental observations. These novel findings suggest the intrinsic ATPase activity of P2RX2 and provide molecular insights into the channel opening.

## Introduction

P2X receptor family comprises seven different receptors, P2RX1 to P2RX7 (Mittal et al., 2016). These receptors are expressed in a wide variety of cell types and are involved in numerous physiological processes, including platelet aggregation, immune responses smooth muscle contraction, inflammation, and sensory neurotransmission (Burnstock, 2013; Zhang et al., 2015; Martínez-Ramírez et al., 2016; Riding and Pullar, 2016; Sáez-Orellana et al., 2016). Out of these seven receptors, P2RX2 is an ATP-gated trimeric ion channel that plays an important role in sound transduction and auditory neurotransmission in the inner ear (Housley et al., 2002, 2013; Järlebark et al., 2002; Wang et al., 2003; Yan et al., 2013). ATP binding to the extracellular loop of the channel is thought to cause conformational changes that trigger channel pore opening and cation internalization (North, 2002; Roberts et al., 2006; Stelmashenko et al., 2012; Mittal et al., 2016; Wang and Yu, 2016). However, it is not known whether ATP binding or hydrolysis is required for the P2X2 activation.

Ion channels including ATP-gated P2RX2 channels have been demonstrated to be regulated by phosphoinositides (PIP _*n*_s) (Hille et al., 2015). PIP _*n*_s are minor phospholipids that comprises less than 1% of total membrane lipids, but play a very crucial role in cell signaling events (Viaud et al., 2015; Waugh, 2015; Marat and Haucke, 2016). PIP _*n*_s are involved in the activation of many ion channels and enzymes, and regulate virtually all membrane trafficking events, including endocytosis and exocytosis (Swanson, 2014; Levin et al., 2015; Posor et al., 2015). PIP _*n*_s also recruit proteins to the plasma membrane or intracellular compartments through several structured interaction domains (Balla, 2013; Cauvin and Echard, 2015). The pharmacological depletion of PIP _*n*_s with the PI3K blockers wortmannin and LY294002 has been demonstrated to affect P2RX2 channel gating (Fujiwara and Kubo, 2006). The lack of PIP _*n*_s accelerated P2RX2 channel desensitization that was also observed with two mutations, K365Q or K369Q in the conserved, positively charged, amino acid residues in the proximal region of the cytoplasmic C-terminal domain. These findings suggest that the interaction between lysine residues at positions 365 and 369 with PIP _*n*_s play an important role in stabilizing the open conformation of the P2X2 channel. It was demonstrated that P2X2 pore dilation is closely linked to channel desensitization and is regulated by the binding of PIP _*n*_s to the cytoplasmic C-terminal region of the channel by determining the time-dependent permeability shift in N-methyl-D-glucamine (NMDG+)- (Fujiwara and Kubo, 2006). GST-tagged recombinant proteins spanning the proximal C-terminal region of P2RX2 were able to directly bind to PIP _*n*_s. EGFP tagged fusion proteins comprising the proximal C-terminal region of P2X2 expressed in COS-7 cells closely associated to the membrane PIP _*n*_s. These results suggest that PIP _*n*_s play a key role in regulating P2X2 channel activity and pore dilation.

To understand the molecular mechanisms underlying opening and closing of P2RX2 through ATP binding, different amino acids have been mutated using site-directed mutagenesis in purified rat P2RX2 (rP2RX2) or human P2RX2 (hP2RX2) (Chataigneau et al., 2013; Jiang et al., 2013; Dal Ben et al., 2015; Habermacher et al., 2016). Mutations namely, F183C, T184C, and F289C causes 4–10-fold decrease in ATP binding to hP2X2 (Roberts et al., 2008; Chataigneau et al., 2013). The mutations N288C, R290C, and K307C have also been implicated in decreased binding of ATP to hP2X2 (Roberts et al., 2008; Chataigneau et al., 2013). Mutations K69C, and K71C leads to non-functional hP2X2 that is unable to bind ATP (Roberts et al., 2008; Chataigneau et al., 2013). It has been demonstrated that two residues N140 and L186 play a crucial role in ATP binding to rP2X2 using a thiol-reactive probe (8-thiocyano-ATP, NCS-ATP) (Jiang et al., 2011).

Some of the mutations in cation channels including P2X2 have clinical implications and have been associated with hearing loss in humans. Hearing loss is the most common sensory deficit in human populations causing significant deterioration in the quality of life (Géléoc and Holt, 2014). About 50–60% of hearing loss cases have a genetic etiology (Ouyang et al., 2009; Angeli et al., 2012; Bogo et al., 2015; Chakchouk et al., 2015; Grati et al., 2015; Parker and Bitner-Glindzicz, 2015; Qing et al., 2015; Wang et al., 2015; Yan et al., 2015). The remaining 40–50% of cases are attributed to environmental factors such as ototoxic drugs, prematurity, or trauma (Roizen, 1999; Furness, 2015; Momi et al., 2015). However, as public health awareness is improved, environmental factors are contributing less to the etiology of deafness and the relative proportion of genetic hearing loss is increasing. Approximately, one in every 1,000 children has some form of prelingual hearing impairment, and one in 2000 is caused by a genetic mutation (Vele and Schrijver, 2008). About 30% of cases of prelingual deafness are classified as syndromic; the remainder cases are nonsyndromic (Stelma and Bhutta, 2014). We and others have demonstrated that V60L and G353R mutations in P2RX2 cause dominant progressive hearing loss in humans (Yan et al., 2013; Faletra et al., 2014). Since these mutations cause deafness and have significant clinical implications, it is worthwhile to examine how these mutations affect the physiological function of P2RX2 cation channel. Understanding the mechanisms through which these mutations affect the normal function and activation of P2RX2 will help in designing novel treatment modalities against hearing loss. Intriguingly, we observed that ATP binding as well as its hydrolysis is an essential step for the hP2RX2 activation. The mutation, V60L, in this cation channel hampers the ability of hP2RX2 for ATP hydrolysis and subsequent activation.

## Materials and methods

### Purification of proteins

Wild-type (WT) and mutant forms of human P2RX2 (hP2RX2) were obtained from Origene (Rockville, MD). Briefly, recombinant proteins were overexpressed in HEK293 cells and then purified using anti-DDK affinity column followed by conventional chromatography steps. The purity of proteins was examined by Coomassie staining. Western blotting was also used to confirm the purity of the proteins employing P2RX2 antibody (Abcam, Cambridge, MA).

### Patch-clamp analysis of ATP-evoked currents

HEK293 cells were cultured in DMEM with 10% fetal bovine serum (FBS) and 100 U/mL penicillin at 37 °C in a 5% CO_2_ incubator. At 90% confluence, cells were passed by trypsin-EDTA, reseeded at a 24-well plate with a density of 100,000 cells per well and incubated overnight. The medium was then replaced with the fresh DMEM plus 10% FBS and a transfection reaction mixture containing OPTI-MEM medium, Lipofectamine 2000, and the *P2RX2* plasmid (WT or mutant forms). After 24–48 h, successful transfectants were identified under fluorescent microscopy. Cells were then trypsinized and replated with normal extracellular solution (130 mM NaCl, 5 mM KCl, 1.47 mM MgCl_2_, 2 mM CaCl_2_, 25 mM dextrose, and 10 mM Hepes; 300 mOsm, pH 7.2) in 35-mm culture dishes for whole-cell patch clamp recordings. Single, isolated transfected HEK293 cells with strong fluorescence were selected, and whole-cell recording was performed. Cells were placed in a recording chamber containing extracellular solution of the following composition (in mM): 140 NaCl, 10 HEPES, 1 MgCl_2_, 2 CaCl_2_, 10 glucose (pH 7.4, ~315 mOsm) and visually identified using a 60 × objective (0.9 numerical aperture) (Olympus) and infrared differential interference contrast. Recording electrodes (2.5–4 MΩ) were pulled from thick-walled borosilicate glass (Sutter Instruments) and filled with intracellular solution that contained (in mM): 140 CsCl, 10 HEPES, 1 MgCl, 5 EGTA (pH 7.3, ~295 mOsm). Data were filtered at 10 kHz using a Multi-clamp 700B amplifier (Molecular Devices) and sampled at 10 kHz. Series resistance (5–12 MΩ) was compensated by 70–80%. Responses were evoked by local puff application via a glass patch-clamp pipette connected to a Picospritzer (Parker Hannifin, Pine Brook, NJ). All data were acquired and analyzed using custom routines written in IgorPro (WaveMetrics). In some experiments we used hydrolysable ATP, non-hydrolysable ATP [adenosine 5′-(β,γ-imido) triphosphate tetralithium salt hydrate, AMP-PNP] or ADP in the concentration range of 36 μM to 1 mM as described in earlier studies (Li et al., 2013; Yan et al., 2013) and recorded current responses.

### ATpase, ADP, and ATP assays

ATP hydrolysis activity of P2RX2 WT and mutant proteins were performed based on a BIOMOL Green method (Harder et al., 1994) (Enzo Life Sciences, Farmingdale, NY). This colorimetric phosphate quantitation method measures free phosphate released to solution. Briefly, 2.5 pmol of purified P2RX2 WT, V60L, G353R, and K81A proteins were incubated with 1 mM of fresh-made ATP in the presence of either NaCl, or KCl, or CaCl_2_ in a MOPS buffer (50 mM MOPS, pH 7.0, 0.1% Triton X-100, 1 mM MgCl_2_, 100 mM indicated salt) for 30 min. In some experiments, different concentrations of P2X2 specific inhibitor, RB-4 (Baqi et al., 2011), were also included. After measuring OD_630_ of the reaction mixture, determination of the released phosphate was calculated as described by the manufacturer (Enzo Life Sciences, Farmingdale, NY). Phosphate standards were used to calibrate a standard curve and ATP hydrolysis activity was expressed as nanomol phosphate released as per manufacturer's instructions. For determination of ADP and AMP levels, proteins were incubated with ATP as described above and then subjected to ADP and AMP assay using kits from Sigma (St. Louis, MO) as per manufacturer's instructions.

### Filter-binding assay

The binding of WT hP2RX2 and its mutant forms to radioactive ATP was determined by Filter- binding assay as described earlier (Makise et al., 2003). Briefly, proteins were incubated with [α-^32^P] ATP or [^3^H] AMP-PNP for 30 min in protein buffer (25 mM Tris, 100mM KCl, 5mM MgCl_2_, 1 mM DTT, pH 7.6). Samples were passed through nitrocellulose membranes (Millipore HA, 0.45 μm) and washed with ice cold protein buffer supplemented with 40 mM MgCl_2_. The radioactivity remaining on the filter was determined with a liquid scintillation counter.

### Rat inner ear explant cultures and transfections

Organs of Corti from inner ears of postnatal day 3 (P3) rats (of either sex) were dissected (Grati et al., 2006; Salles et al., 2009) in accordance with National Institutes of Health guidelines and maintained for 1–2 days in culture. The study protocol was approved by the Institutional Animal Care and Use Committee (IACUC) of the University of Miami. Explants were used to examine the endogenous ATPase activity in the absence or presence of P2RX2 competitive antagonist, RB-4.

### Structural modeling

Structural model of human P2X2 ion channel (residues 41–365) in closed and open states was built using the MODELER software based on homology modeling (Martí-Renom et al., 2000). Briefly, the corresponding crystal structures of the related zebrafish P2RX4 ion channel in open (PDBID 4DW0) and closed (PDBID 4DW1) states were used as templates. It is noteworthy that the zebrafish and human P2RX channels share close to 50% amino acid sequence identity, implying that the structural models of the latter can be relied upon with a high degree of confidence. In each case, a total of 100 atomic models were calculated and the structure with the lowest energy, as judged by the MODELER Objective Function, was selected for further analysis. The structural models were rendered using RIBBONS (Carson, 1991). The closed and open states were morphed with the VMD software (Humphrey et al., 1996).

### Statistical analysis

Results were statistically analyzed using one-way ANOVA with a post hoc test. *P* < 0.05 was considered statistically significant.

## Results

### Electrophysiological recordings

**Figure 1:**
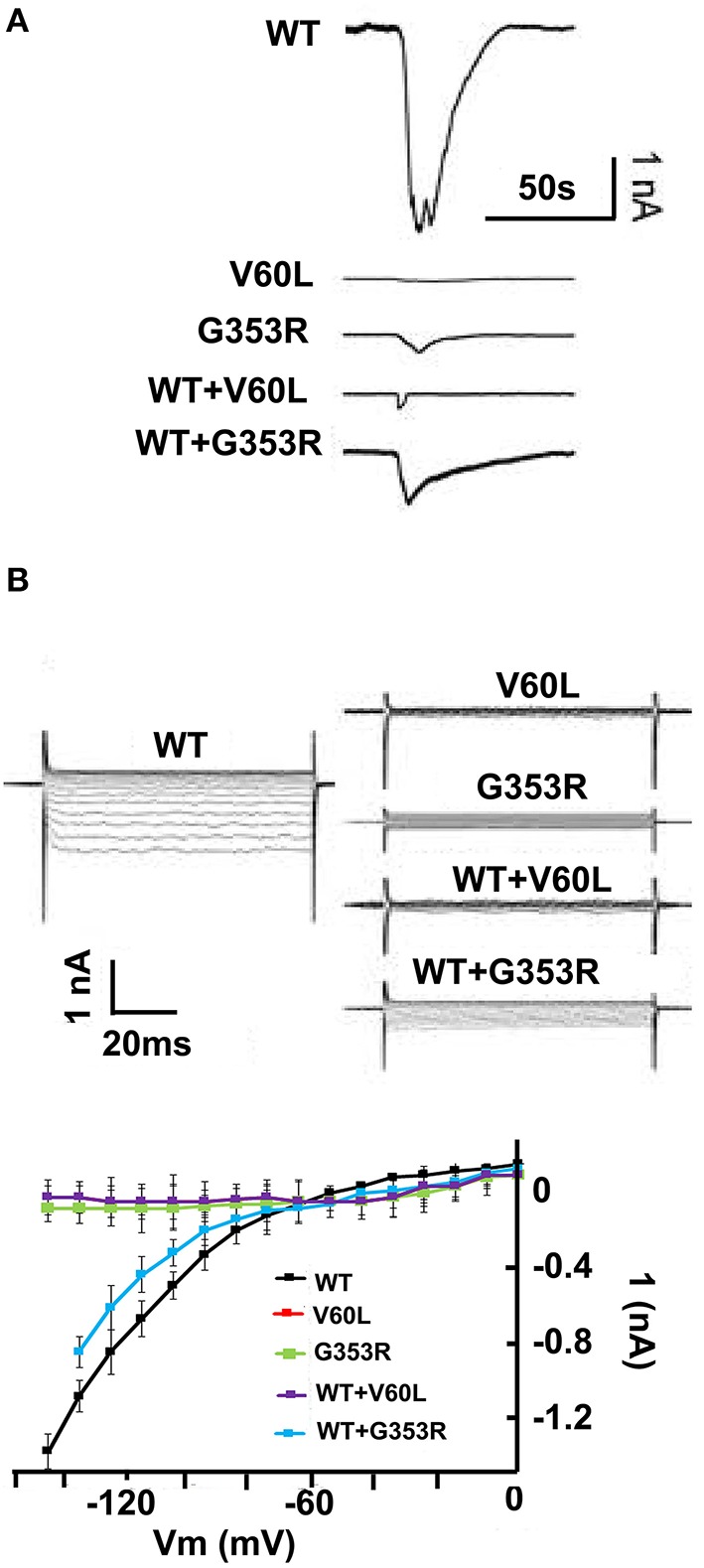
**P2RX2 p.V60L attenuates ATP evoked inward current**. HEK293 cells were transfected with GFP-tagged WT or mutant P2RX2-encoding plasmids and were patch-clamp recorded for inward current following stimulation with ATP (36 μM–1 mM). **(A)** ATP-evoked inward current is visible in an HEK293 cells expressing WT P2RX2 that is abolished in cells expressing mutant P2RX2 V60L. Cells expressing mutant P2RX2 p.G353R show a lower inward current peak than those expressing WT P2RX2. Cells co-expressing WT and mutant P2RX2 showed attenuated inward current compared to those expressing only WT P2RX2. **(B)** The inward rectifying ATP-gated current across the applied voltage range in an HEK293 cells expressing WT or mutant P2RX2. The current–voltage (I-V) relationship was determined as the average current over the last 20 ms at the various voltage steps during the steady-state phase of the ATP-gated inward current. Whole-cell recording was performed using an Axopatch 200B patch clamp amplifier (Molecular Devices) and data were analyzed with jClamp. The experiments were performed three different times in triplicate. Error bars indicate standard deviations.

Using patch clamp recording, ATP stimulation (concentration ranging from 36 μM to 1 mM) of HEK293 cells expressing WT P2RX2 evoked a large inward current, as did to a lesser intensity G353R form, but a very faint or no current was obtained with V60L form (Figures 1A,B).

### Purification of proteins and ATPase activity

**Figure 2:**
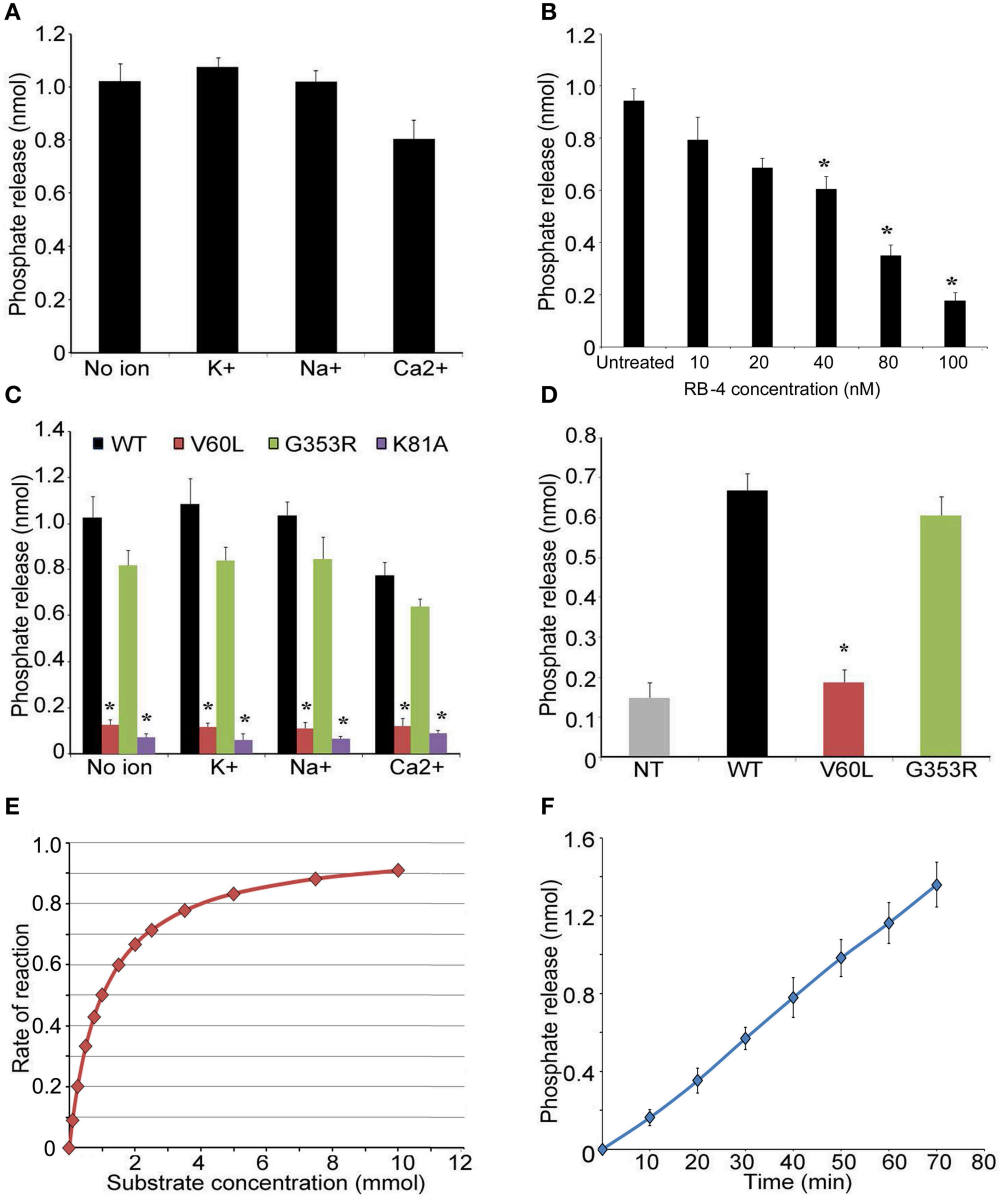
**P2RX2 possesses ATPase activity. (A)** ATP hydrolysis by purified P2RX2 protein was determined in the presence or absence of diverse cations by BIOMOL green reagent. **(B)** In some experiments, P2RX2 specific competitive antagonist RB-4, was included in the reaction mixture. RB-4 was able to reduce the ability of P2RX2 to hydrolyze ATP in a dose dependent manner. **(C)** The effect of mutations in P2RX2 was evaluated in the absence or presence of diverse cations. **(D)** ATP hydrolysis was measured in live HEK293 cells expressing WT, V60L, and G353R P2RX2. **(E)** Michaelis-Menten kinetics equation was used to determine the K_m_ and V_m_ of P2RX2 ATPase activity. **(F)** Plot of ATP hydrolysis as a function of incubation time. The experiments were performed three different times in triplicate. Error bars indicate standard deviations. ^*^P < 0.01 by one-way ANOVA with *post-hoc* test.

Next, we overexpressed and purified P2RX2 and its mutant forms in HEK293 cells. We also used in this study P2RX2 K81A mutant form which has previously proven incapable of binding ATP (Jiang et al., 2000; Wilkinson et al., 2006). The purity of the proteins was determined by SDS page, Commassie staining and Western blotting (Figures S1A,B). Purified proteins were incubated with ATP and phosphate release was monitored using Biomol Green reagent (Enzo Life Sciences, PA). As P2RX2 is a cation channel that regulates the influx of ions including K^+^, Na^+^, and Ca^2+^, we determined the phosphate release in the presence of theses ions. P2RX2 was found to actively hydrolyze ATP and is independent of the presence of K^+^, Na^+^, or Ca^2+^ (Figure 2A). This ATPase activity was significantly inhibited by P2RX2 competitive antagonist, RB-4, confirming the specificity of the reaction (Figure 2B). Interestingly, P2RX2 V60L's ability to hydrolyze ATP was significantly attenuated compared to WT or G353R P2RX2 (*P* < 0.001; Figure 2C). To further confirm these findings, HEK293 cells were transfected with WT and mutant forms of P2RX2 and ATP hydrolysis was determined in live cells. We observed that HEK293 expressing V60L P2RX2 released significantly less inorganic phosphate (iP) than WT or G353R P2RX2 (*P* < 0.001; Figure 2D). Using Michaelis-Menten kinetics equation, we calculated *Km* value for ATP and found it to be 0.62 mM, indicating high ATP affinity (Figure 2E). Following first order reaction kinetics, ATP hydrolysis was linearly proportional with reaction time (Figure 2F).

### Non-hydrolysable ATP failed to evoke inward current responses

**Figure 3:**
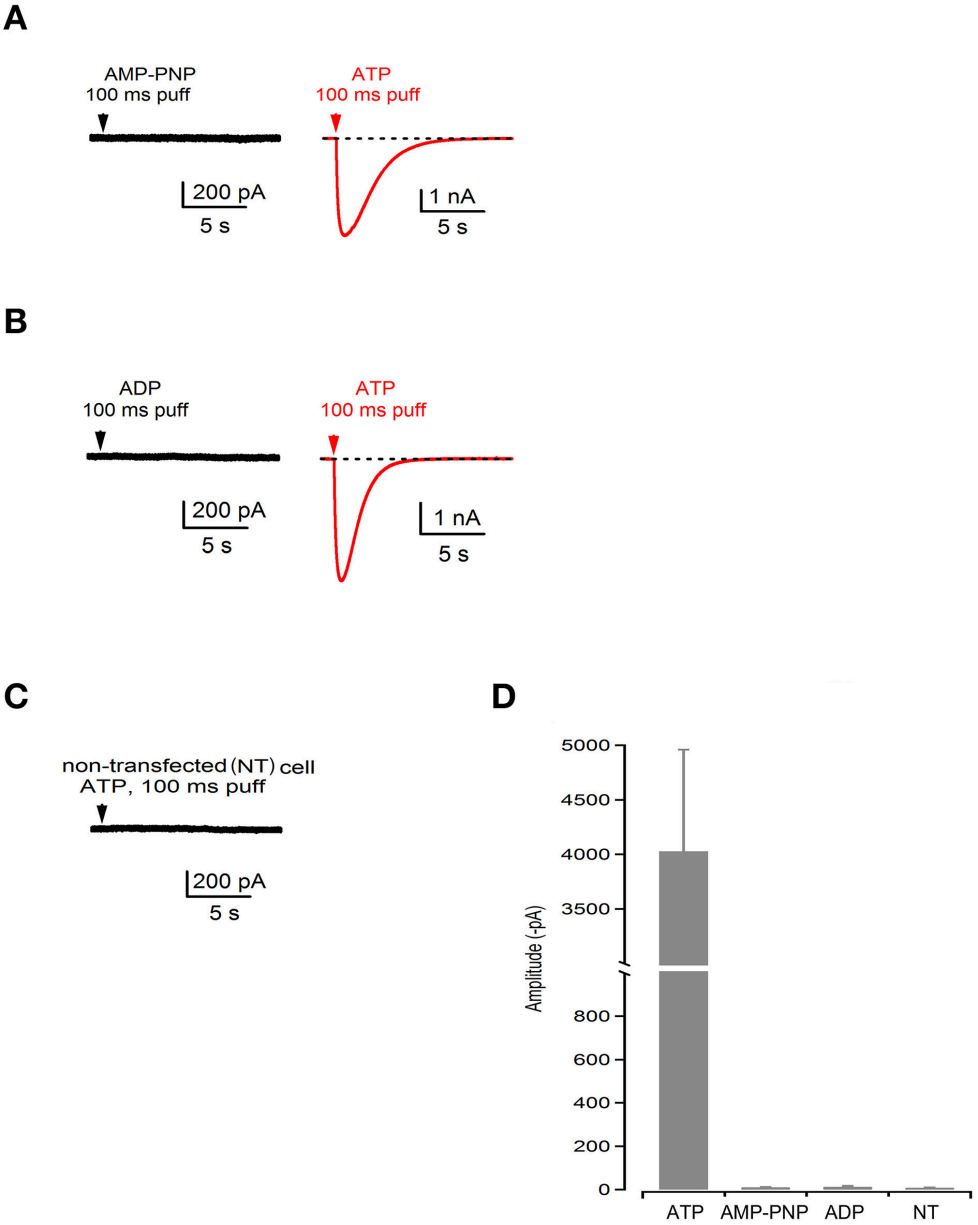
**ATP hydrolysis plays a crucial role in gating of P2RX2 channel. (A–C)** Current recording during application of a nonhydrolysable analog of ATP (AMP-PNP) or ADP followed by the application of hydrolysable ATP (red traces) performed in the same cell or in non-transfected (NT) cells. **(D)** Summary bar graph of amplitudes during application of ATP, AMP-PNP, and ADP in transfected and non-transfected cells.

We further performed electrophysiological recordings on HEK293 cells to investigate the gating properties of WT P2XR2 ion channels. We locally applied hydrolysable ATP, non-hydrolysable ATP analog [adenosine 5′-(β,γ-imido) triphosphate, AMP-PNP] or ADP, ranging in concentration from 36 μM to 1 mM, and recorded current responses. Our results show that local application of hydrolysable ATP for 100 ms evoked large responses (−4029 ± 931 pA, *n* = 6 cells) mediated by P2RX2. On the contrary, application of AMP-PNP, or ADP did not evoke any responses (−11 ± 1 pA for AMP-PNP, *n* = 6 cells; −13 ± 4 for ADP, *n* = 5 cells) and the peak values analyzed as minimal peaks from the zero baseline represent the negative portion of the noise in our recordings (Figures 3A,B,D). Cells that were not responsive to AMP-PNP or ADP were still responsive to hydrolysable ATP (Figures 3A,B,D). We used higher concentrations of ATP and AMP-PNP (up to 1mM) for electrophysiological recordings to determine if saturating the P2RX2 receptor with non-hydrolysable analog can open the channel. However, even at higher concentrations, AMP-PNP failed to invoke inward current responses. Further, we did not observe any current responses from non-transfected cells following stimulation with higher ATP concentrations ruling out the contribution of other P2RX2 receptors at higher concentrations (Figures 3C,D). In agreement with these results, we did not observe any current responses from HEK 293 cells transfected with G353R P2RX2 stimulated with non-hydrolysable AMP-PNP or ADP (data not shown). From these results, we concluded that ATP hydrolysis plays a crucial role in opening the P2RX2 ion channels.

### ATPase activity of cochlear neuroepithelium expressing endogenous P2rx2

**Figure 4:**
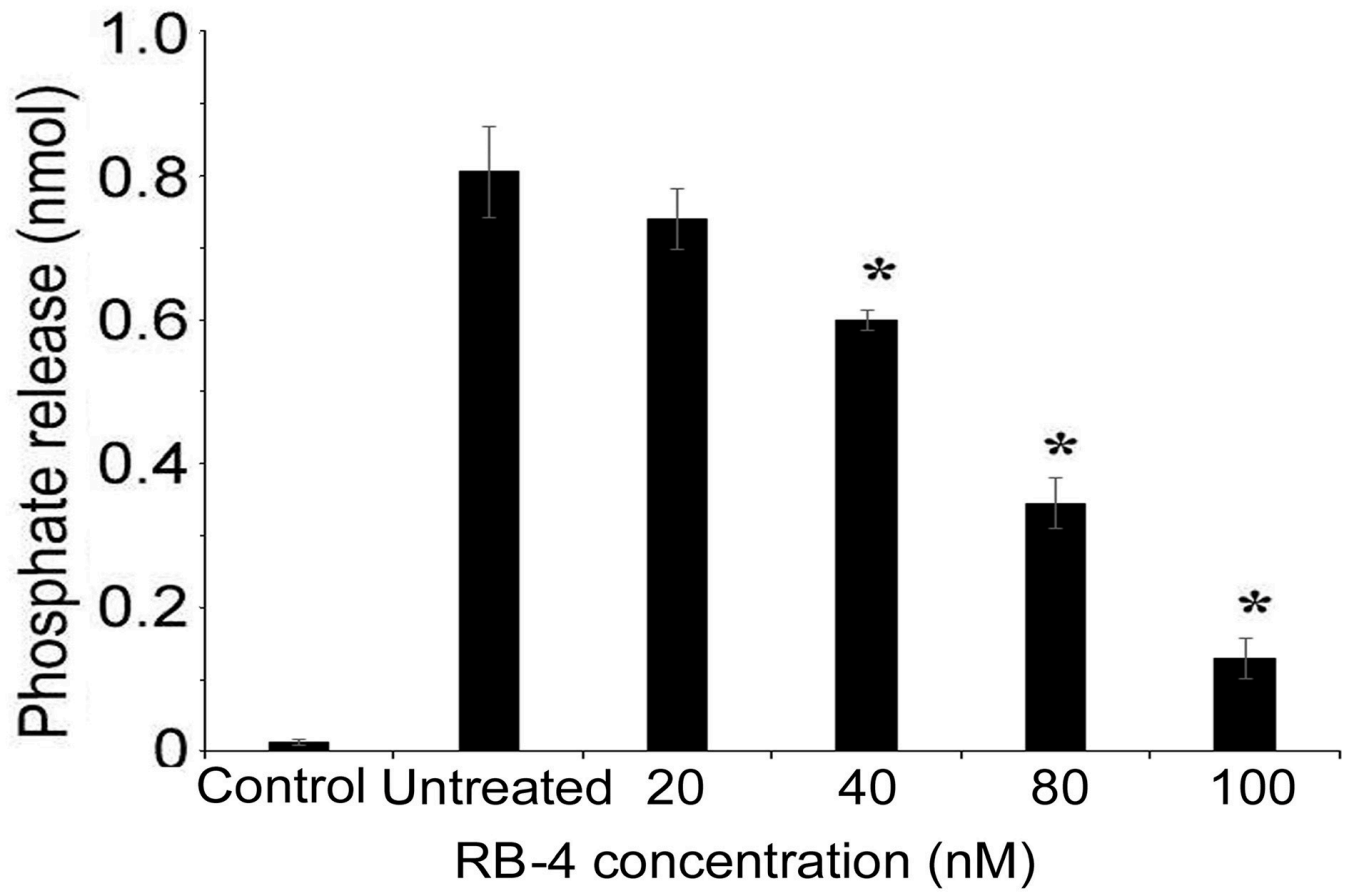
**ATPase activity of organ of Corti explant cultures (A) Cochlea explant cultures were established from 3 day old rats and incubated with ATP**. The hydrolysis of ATP was then examined using BIOMOL green reagent. **(B)** ATPase activity of the cochlear organotypic explant cultures expressing endogenous P2RX2 was determined in the presence of different concentrations of P2RX2 specific competitive antagonist, RB-4. The results are representative of three different experiments carried out in triplicate. ^*^P < 0.01 by one-way ANOVA with *post-hoc* test.

As P2rx2 plays an important role in the hair cell physiology (Housley et al., 2002, 2013; Järlebark et al., 2002; Wang et al., 2003), we examined ATP-evoked iP-release from postnatal day 3 rat cochlea organotypic culture hair cells that endogenously express P2rx2 (Figure 4). The experiments performed without explant cultures served as control group to rule out the non-specific ATP hydrolysis. The ATPase activity of the developing cochlear neuroepithelium endogenously expressing WT P2rx2 was significantly inhibited in the presence of P2rx2 competitive antagonist RB-4, in a concentration-dependent manner (*P* < 0.01; Figure 4).

### P2RX2 is a classical ATPase and V60L mutation hampers its ability to bind ATP

**Figure 5:**
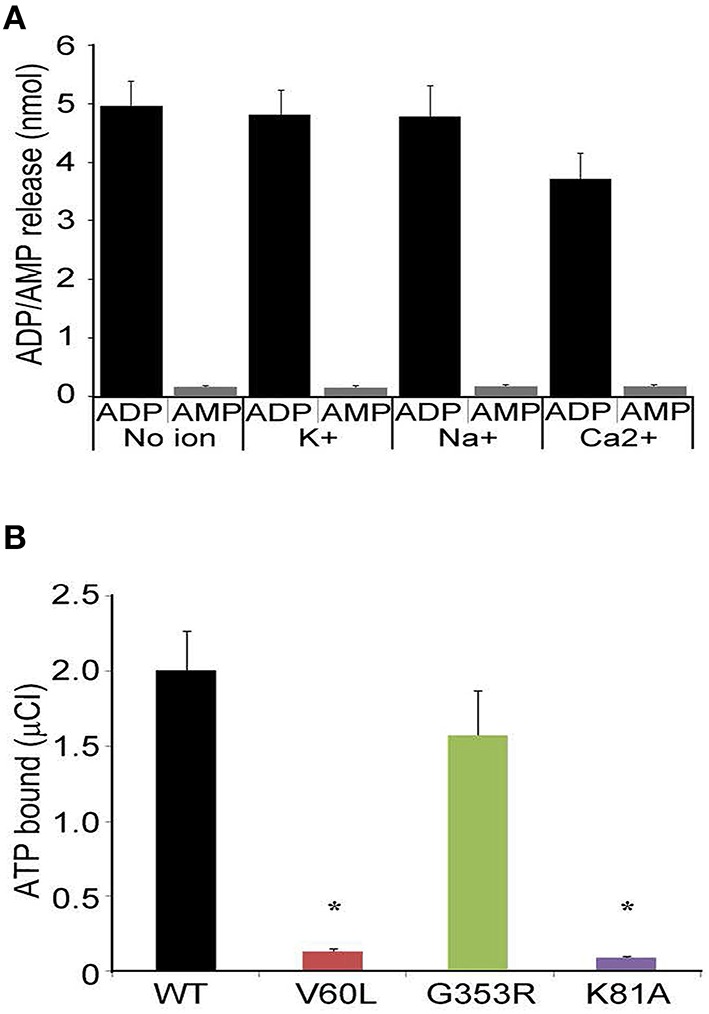
**ADP/AMP and radioactive ATP binding assays. (A)** Purified P2RX2 was incubated with ATP and release of ADP and AMP was determined in the absence or presence of diverse cations using ELISA kits (Sigma). **(B)** P2RX2 wild-type as well as mutant V60L, G353R, and K81A forms were incubated with radioactive ATP, and their ATP binding capabilities were determined using the filter binding assay as described in Materials and Methods section. The results are representative of three different experiments carried out in triplicate. Error bars indicate standard deviations. ^*^P < 0.01 by one-way ANOVA with *post-hoc* test.

Next we determined whether P2RX2 is a classical ATPase releasing ADP as a final product or ATP hydrolyzer with AMP as a final product. To examine this, we incubated purified protein with ATP and determined ADP or AMP release using commercially available kits. We observed that P2RX2 is indeed a classical ATPase that catalyzes the conversion of ATP into ADP but not to AMP (Figure 5A).

It is possible that the inability of P2RX2 V60L to hydrolyze ATP is due to the lack of ATP binding. To determine this, we performed radioactive ATP filter binding assays for all four purified forms of P2RX2. WT and G353R P2RX2 bound to radioactive ATP proficiently whereas V60L and K81A P2RX2 forms showed significantly attenuated ATP binding activity (*P* < 0.001; Figure 5B). These results suggest that the V60L mutation hampers the ability of P2RX2 to bind ATP and hence abolishes its ATPase activity.

### Computer modeling suggests that P2RX2 V60L mutation affects the conformation of P2RX2 leading to altered ATP binding and subsequent hydrolysis

**Figure 6:**
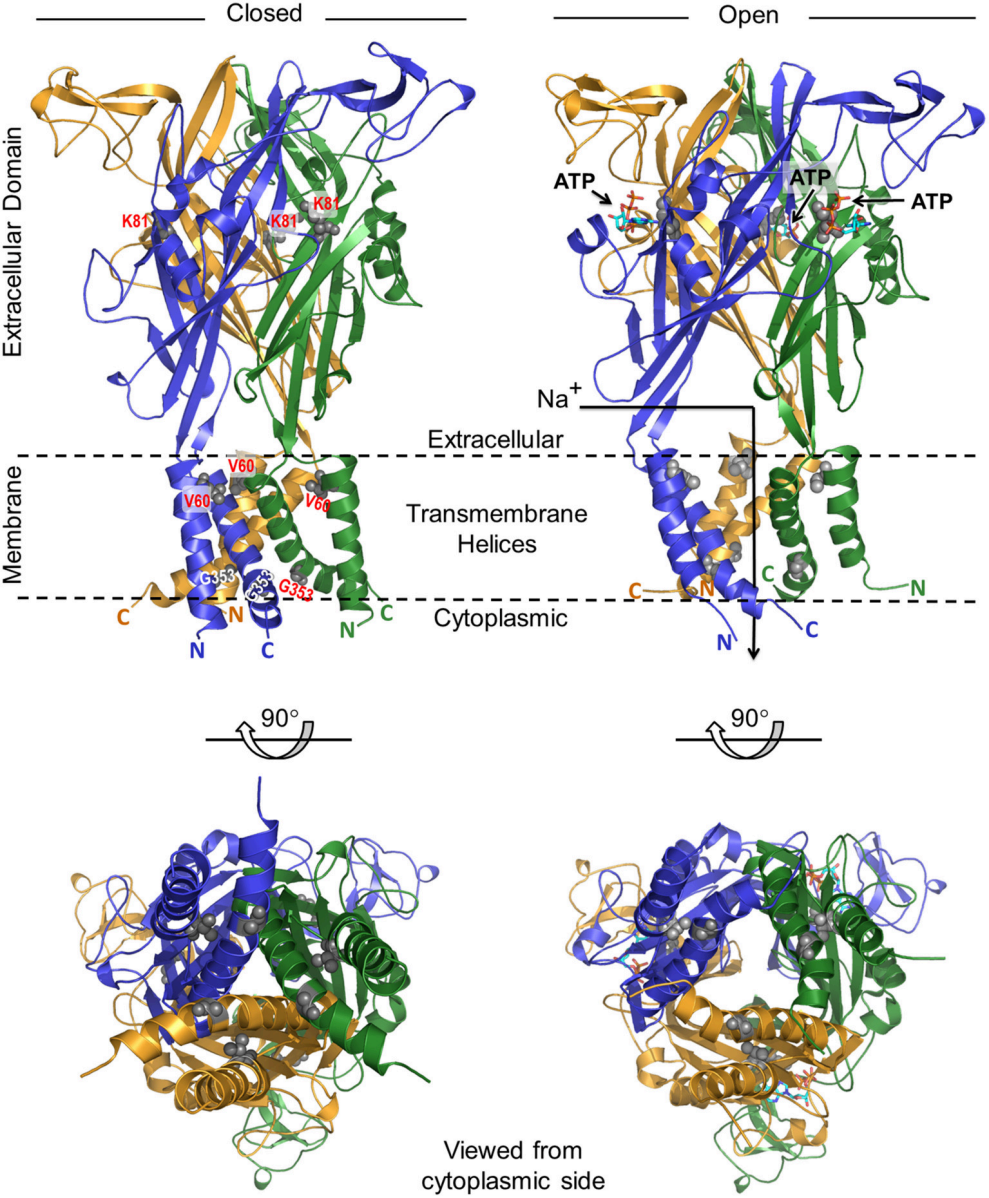
**Ribbon representation of the structural model of human P2RX2 ion channel in the closed and open states**. In each case, two alternative orientations of the P2RX2 channel related by a 90°-clockwise rotation about the horizontal axis are displayed. Within each orientation, the homotrimeric P2RX2 channel is color-coded with the three monomers shown in yellow, blue, and green. Additionally, the side chain moieties of V60 and G353 residues located within the transmembrane helices and K81 within the extracellular domain are all shown in red. The numerals 41 and 365 respectively indicate the N-terminal and C-terminal residue boundaries of the modeled region of P2RX2 channel and the parenthesized letters following these numerals represent each of the three monomers. The arrow traversing the ion channel in the open state denotes the route of the influx of cations such as Na^+^ upon channel opening. Residue atoms for V60, K81, and G353 are highlighted as gray spheres. The three ATP molecules are shown as sticks.

To understand the molecular basis of how V60L, K81A, and G353R mutations affect the physiological function of the P2RX2 channel, we modeled its three-dimensional structure in both closed and open states spanning residues 41–365. As presented in Figure 6, our structural analysis shows that the P2RX2 channel adopts a canonical trimeric fold with a three-fold axis of symmetry. V60L mutation can result in altered conformational changes leading to inability of P2RX2 channel to bind ATP and subsequently ATP hydrolysis. On the other hand, G353R mutation does not have much impact on the ATP binding and hydrolysis property of P2RX2 and would likely affect the intrinsic properties of the pore ionic permeation.

## Discussion

P2RX receptors are purinergic ligand-gated trimeric ion channels that are widely distributed in mammalian tissues, where they are involved in a range of processes, from inflammation to excitatory synaptic transmission (Puchałowicz et al., 2014; Samways et al., 2014; Xu and Khakh, 2014; Dal Ben et al., 2015; Di Virgilio, 2015). It comprises a family of seven receptors designated P2RX1 to P2RX7 (North, 2002). Out of these seven receptors, P2RX2 plays an important role in auditory and nervous systems (Housley et al., 2002, 2013; Järlebark et al., 2002; Wang et al., 2003; Mittal et al., 2016). Earlier studies have demonstrated that mutations in P2RX2, namely V60L and G353R, lead to hearing loss, demonstrating the importance of this receptor in the inner ear (Yan et al., 2013; Faletra et al., 2014).

The binding of ATP to P2RX2 has been shown to play an important in gating the channel (Roberts et al., 2006). However, it is still not known how P2RX2 acquires the energy required to open the channel and perform its physiological function. In this study, we demonstrate for the first time that P2RX2 is indeed an ATPase that can degrade ATP, leading to the release of ADP and free inorganic phosphate. We conducted these experiments on solubilized purified P2RX2 protein that corroborates with the findings of the earlier studies demonstrating ATPase activity by purified proteins (Gresser et al., 1984; Liu et al., 1997; Nikaido et al., 1997; Li and Altman, 2001; Andrade et al., 2007; Yoshida et al., 2014). We found that P2RX2 is able to catalyze the degradation of ATP in a linear, time dependent manner with a *Km* value of 0.62 mM. We also observed that it only catalyzes the conversion of ATP to ADP and not further to AMP indicating that P2rx2 is an ATPase and not ATP hydrolyzer. Interestingly, V60L mutation abolished its ability to bind ATP and subsequently ATP degradation. The inner ear organ of Corti explant cultures from newborn rats also confirmed the presence of a P2rx2-related ATPase activity.

Protein expression in live cells helps to examine their physiological role in a membrane-associated native conformation. Therefore, we used HEK293 live cells expressing exogenous recombinant WT and mutant forms of P2RX2 and determined their respective ATPase activity. In agreement with the *in vitro* data obtained on purified proteins, we observed a significant release of inorganic phosphate by cells expressing WT or G353R form of P2RX2, but very little or no release by V60L transfected and non-transfected cells, respectively. The experiments were only performed on live HEK293 cells and not on life mammalian neuroepithelia because of (i) the non-availability of the appropriate animal models for each of the studied mutations endogenously expressing mutant P2rx2, and (ii) it is difficult to achieve a significantly high and reproducible transfection and expression rates of the mutant proteins in explant epithelium hair cells to allow the performance of similar measurements. We are in the process of generating mouse knock-in models for each of P2rx2 V60L and G353R mutations, and their respective cochlea neuroepithelia will be used for further investigating endogenous mutant P2rx2 ATPase activity.

The competitive antagonists or inhibitors are useful tools to characterize the activity of target proteins and their specificity. Therefore, we used P2RX2 specific inhibitor RB4, to confirm the ATPase activity of P2RX2. RB-4 is a selective P2RX2 inhibitor that exhibits no activity against other P2RX receptors (Baqi et al., 2011). The ATPase activity of P2RX2 was significantly decreased in the presence of RB-4 inhibitor demonstrating the specificity of ATP degradation by P2RX2. Since we observed significant decrease in ATPase activity with purified P2RX2 protein, it suggests that this activity is contributed only by P2RX2 and not by the other P2RX receptors. However, we observed that EC50 value varies from few to 30 uM, whereas Km for the ATP hydrolysis was about 700 uM. In other words, almost complete channel's activation occurred, when the occupancy of sites, responsible for the ATP hydrolysis, could be low. It is possible that since P2RX2 is a trimeric receptor, binding of few ATP molecules and subsequent hydrolysis is sufficient to trigger channel activation. Further studies are warranted to characterize the relationship between EC50 and Km values for P2RX2.

The generation of inward current in response to ATP is a reliable and well established parameter for monitoring the gating of P2RX and other ion channels (Li et al., 2013; Yan et al., 2013). Our electrophysiology data suggests that ATP degradation is required for opening the P2RX2 channel as ATP non-hydrolysable analog failed to evoke inward currents. It is noteworthy that there are two non-hydrolyzable analogs of ATP, ATPγS, and AMP-PNP. ATPγS has been shown to possess the agonist activity and can activate numerous P2 receptors including P2RX2 (Thomas et al., 1991; Evans et al., 1995). On the contrary, AMP-PNP have been demonstrated to exert antagonist effect on the ATPase activity of many ATPases (Gresser et al., 1984; Liu et al., 1997; Li and Altman, 2001). Therefore, we used AMP-PNP in this study and not ATPγS. Interestingly, AMP-PNP was able to bind purified P2RX2 protein as determined by radioactive filter binding assays (data not shown). This suggests that lack of generation of inward currents in the presence of AMP-PNP is not due its inefficient binding. It would also be possible that ATP hydrolysis occurs after the channel is gated and entered a secondary conformation. In this case, we should get some inward currents with AMP-PNP. However, even by saturating the P2RX2 receptors with higher concentrations of AMP-PNP, we do not observe any inward current responses. This data suggests that ATPase activity plays a crucial role in the early stages of P2RX2 channel opening.

Computer modeling showed that P2RX2 is comprised of a six-helical transmembrane bundle (with a pair of helices contributed by each of the three monomers) hooked onto a predominantly mixed αβ extracellular domain. This domain also harbors the ATP-binding-pocket, in a manner akin to that observed for the zebrafish P2RX4 channel (Kawate et al., 2009; Hattori and Gouaux, 2012). Importantly, comparison of the closed and open states reveals that ATP binding to the extracellular domain is accompanied by a dramatic conformational change within the P2RX2 channel. Though transmitted throughout the channel, these extensive structural rearrangements are particularly concentrated around the transmembrane pore and the adjacent extracellular fenestrations. More specifically, binding of ATP appears to be coupled to radial expansion (along an axis parallel to the membrane surface) of the fenestrations so as to facilitate the diffusion of extracellular cations into the channel pore beneath.

How can we rationalize the functional effects observed *in vitro* for the V60L, K81A and G353R mutations in the context of opening and closing of the P2RX2 ion channel? Interestingly, all three of these mutations map to key regions within the channel. While the K81A mutation lines the ATP-binding pocket within the extracellular domain, the V60L and G353R changes are located within the transmembrane helices. Given that V60 residue is located within the transmembrane pore, close to its extracellular opening, we believe that it plays a key role in relaying and transducing the mechanical movement of the fenestrations into the pulling action exerted on the transmembrane helices so as to release the mechanical energy. While V60L mutation alone may not appear damaging, it is nonetheless likely to alter the dynamics of the whole system, albeit in a subtle manner. Importantly, the triple effect of the V60L mutation due to its presence within each of the three monomers will be expected to be highly cooperative and such synergism could indeed substantially affect the transfer of movement from the expansion of the upstream fenestrations to the opening of the downstream channel pore. Thus, failure to couple such mechanical signal would have a reciprocating effect in that it would resist the fenestrations from undergoing expansion. The subsequent lack of ensuing flexibility and the sustained rigidity of the extracellular domain would in turn be expected to directly affect the formation of the ATP-binding pocket and hence the binding of ATP itself.

While glycine residues tend to destabilize helices in water-soluble proteins, they play a fundamental role in the formation of transmembrane helices (Javadpour et al., 1999; Dong et al., 2012). In particular, glycines induce kinks in transmembrane helices (Video S1) that allow their tight packing and, consequently, promote transmembrane association and oligomerization of polytopic membrane proteins. In light of this knowledge, the G353 residue is thus critical to the intermittent closing and opening of the P2RX2 channel in response to ATP binding. Accordingly, the G353R mutation would be expected to disrupt the native packing of transmembrane helices, thereby resulting in the disruption of ion flow across the P2RX2 channel in remarkable agreement with our *in vitro* data. In summary, our structural analysis of the P2RX2 channel provides an exquisite peek into its dynamics and corroborates our *in vitro* data.

To our knowledge, this is the first study to demonstrate the ATPase activity of P2X2 ion channel. Future investigations will aim to determine key residues that are directly involved in the ATPase catalytic site of the channel, and to exploit these structural and functional insights toward the rational design of novel activators and inhibitors of the P2RX2 channel.

## Author contributions

RM, MG, MS, DY, BK, AF, PC, YZ, and XZL conceived and designed the study. RM, MG, MS, FY, QC, XL, and YZ performed the experiments and analyzed the data. RM, MG, MS, DY, AF, QC, XL, YZ, and XZL wrote the manuscript. All the authors read and approved the final version of the manuscript.

### Conflict of interest statement

The authors declare that the research was conducted in the absence of any commercial or financial relationships that could be construed as a potential conflict of interest.
