# Supplementary figures and images for: Characterization of ATPase Activity of P2RX2 Cation Channel
Source: Front Physiol. 2016 May 24;7:186. doi: 10.3389/fphys.2016.00186 (PMC4878533; doi:10.3389/fphys.2016.00186)

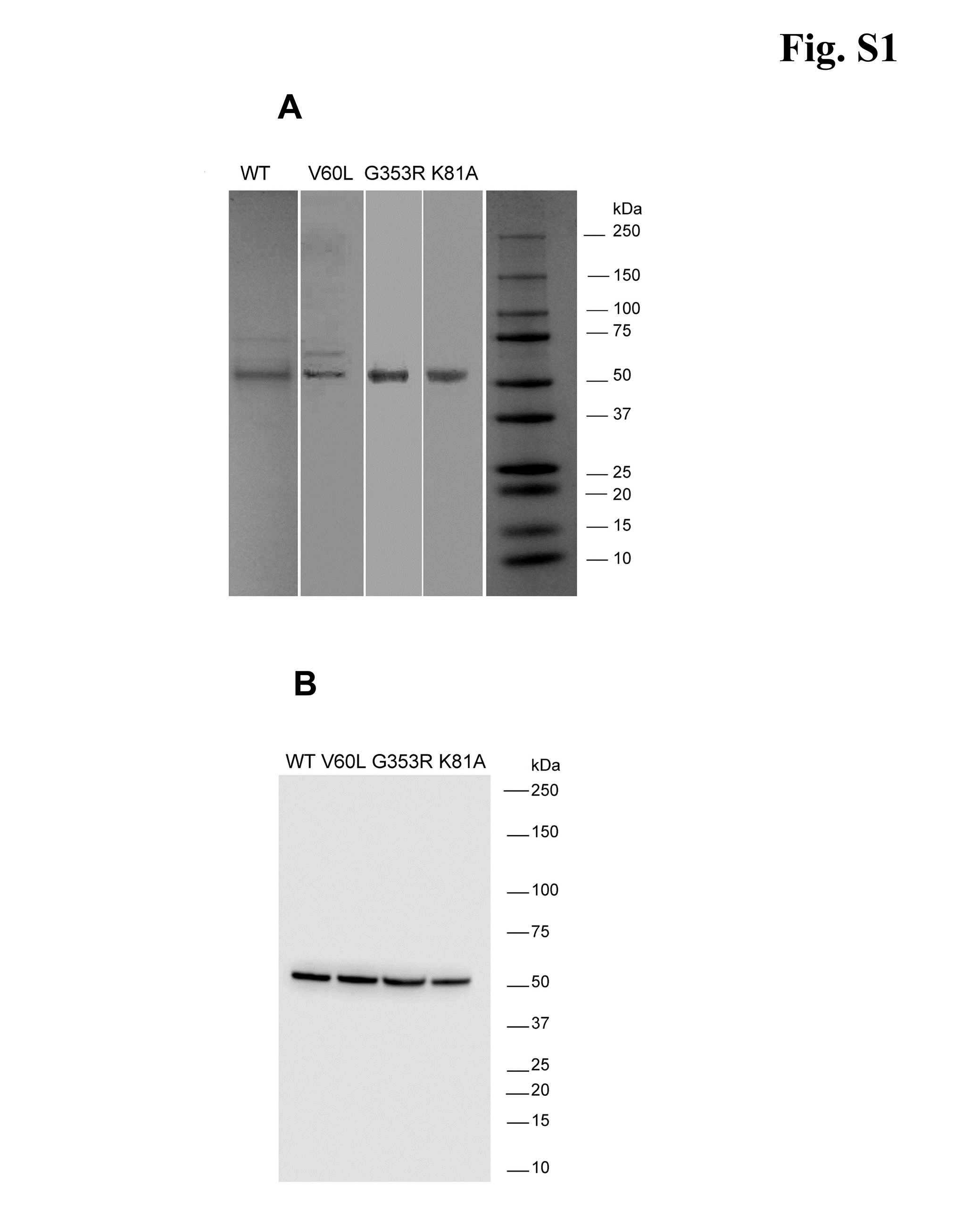

Supplement: Supplementary file 2 [file Image1.TIF]
