# Supplementary material for: Characterization of ATPase Activity of P2RX2 Cation Channel
Source: Front Physiol. 2016 May 24;7:186. doi: 10.3389/fphys.2016.00186 (PMC4878533; doi:10.3389/fphys.2016.00186)
Supplement: Supplementary file 3 [file DataSheet1.PDF]

## Supplemental Figure and Video Legends

**Fig. S1. Purification of P2RX2 proteins.** (A) Proteins were analyzed by SDS-page and stained with Commassie brilliant blue. (B) Western blotting was used to evaluate the purity of proteins.

**Video S1**

Animation demonstrating the effect of P2RX2 mutations on its ability to bind ATP. Mutations in P2RX2 affect its ability to bind ATP and to undergo conformational changes required for channel function. Structural modelling showed that the V60L mutation causes a conformational change affecting the ATP binding pocket of P2RX2. On the other hand, G353R disrupts the native packing of transmembrane helices leading to disruption of ion flow across the P2RX2 channel without affecting its ability to bind ATP.
